# Supplementary material for: B.Y.O. Bees: Managing wild bee biodiversity in urban greenspaces
Source: PLoS One. 2023 Apr 26;18(4):e0281468. doi: 10.1371/journal.pone.0281468 (PMC10132636; doi:10.1371/journal.pone.0281468)
Supplement: S1 Table — Summary of site bee abundance, richness and sampling effort. (DOCX) [file pone.0281468.s001.docx]

**S1 Table.** Summary of site bee abundance, richness and sampling effort.

| **Site Code** | **Site Full Name** | **Management Classification** | **Number of sampling events** | **Total Number of Bees Collected** | **Total Site Species Richness** |
| --- | --- | --- | --- | --- | --- |
| BUBF | Bubolz Nature  Preserve Wet Meadow | Managed  Suburban | 8 | 67 | 20 |
| BUBH | Bubolz Nature Preserve Prairie | Managed Suburban | 8 | 97 | 23 |
| CITY | City Park | Unmanaged  Urban | 8 | 21 | 8 |
| HKRDT | Heckrodt Wetland Reserve- Prairie | Managed Urban | 8 | 102 | 24 |
| LCKR | Lecker County Park | Managed Suburban | 8 | 137 | 21 |
| MAIN | Lawrence University- Main Hall Lawn | Unmanaged Urban | 8 | 31 | 11 |
| MEMPK | Memorial Park | Managed  Urban | 8 | 166 | 29 |
| PBDY | Peabody Park | Unmanaged Urban | 8 | 24 | 11 |
| PRCPK | Pierce Park | Unmanaged  Urban | 8 | 22 | 9 |
| PRDYF | Purdy Nature Preserve- Prairie | Managed Suburban | 8 | 214 | 27 |
| PRDYF | Purdy Nature Preserve- Mowed Lawn | Managed Suburban | 8 | 223 | 30 |
| RVWH | Riverview Gardens Prairie | Managed Urban | 8 | 95 | 20 |
| RVWF | Riverview Gardens  Mowed Lawn | Unmanaged  Urban | 8 | 45 | 18 |
| SLUG | Lawrence  University Sustainability Garden | Managed Urban | 8 | 140 | 28 |
| TLHPK | Telulah Park | Unmanaged Urban | 8 | 34 | 16 |
| TISL | Thousand Islands  Environmental Center | Managed  Urban | 8 | 97 | 25 |
|  |  |  | **Total Bee Abundance & Richness** | 1516 | 86 |
