# Supplementary material for: B.Y.O. Bees: Managing wild bee biodiversity in urban greenspaces
Source: PLoS One. 2023 Apr 26;18(4):e0281468. doi: 10.1371/journal.pone.0281468 (PMC10132636; doi:10.1371/journal.pone.0281468)
Supplement: S2 Table — Summary of total abundances of all wild bee species collected across all sites. (DOCX) [file pone.0281468.s002.docx]

**Table 2:** Summary of total abundances of all wild bee species collected across all sites.

| Species | Total Count |
| --- | --- |
| **Andrenidae** | |
| *Andrena biseleasis* | 9 |
| *Andrena crataegi* | 4 |
| *Andrena cressoni* | 8 |
| *Andrena forbesi* | 1 |
| *Andrena hippotes* | 2 |
| *Andrena jermindus* | 1 |
| *Andrena miranda* | 25 |
| *Andrena nasoni* | 3 |
| *Andrena ostenis* | 2 |
| *Andrena wilkea* | 3 |
| **Apidae** | |
| *Apis mellifera* | 43 |
| *Bombus bimaculatus* | 6 |
| *Bombus citrinus* | 1 |
| *Bombus fervidus* | 2 |
| *Bombus griseocollis* | 6 |
| *Bombus impatiens* | 27 |
| *Bombus rufocinctus* | 2 |
| *Bombus ternarius* | 2 |
| *Bombus vagans* | 32 |
| *Ceratina calcarata* | 36 |
| *Ceratina mikmaqi* | 3 |
| *Melissodes agilis* | 1 |
| *Melissodes bimaculatus* | 64 |
| *Melissodes denticulatus* | 8 |
| *Melissodes desponsus* | 78 |
| *Melissodes druinellus* | 22 |
| *Melissodes rustica* | 4 |
| *Melissodes subillatus* | 3 |

| *Nomada cressoni* | 4 |
| --- | --- |
| *Peponapis pruinosa* | 2 |
| Species | Total Count |
| **Colletidae** | |
| *Hylaeus annulatus* | 1 |
| *Hylaeus hyalinantus* | 3 |
| *Hylaeus modestus* | 5 |
| *Hyleaus affinis* | 1 |
| *Hyleaus mesillae* | 4 |
|  | |
| **Halictidae** | |
| *Agapostemon sericeus* | 1 |
| *Agapostemon splendens* | 1 |
| *Agapostemon virescens* | 54 |
| *Augochloropsis n. falgida* | 1 |
| *Augochorella aurata* | 57 |
| *Augochorella pura* | 5 |
| *Dufourea monardae* | 1 |
| *Halictus confusus* | 10 |
| *Halictus ligatus* | 5 |
| *Halictus rubicundus* | 150 |
| *Lasioglossum absimile* | 3 |
| *Lasioglossum anomalum* | 70 |
| *Lasioglossum bruneri* | 1 |
| *Lasioglossum cinctipes* | 4 |
| *Lasioglossum coeruleum* | 19 |
| *Lasioglossum coriaceum* | 14 |
| *Lasioglossum cressonii* | 425 |
| *Lasioglossum ellsiae* | 1 |
| *Lasioglossum heterognathum* | 15 |
| *Lasioglossum hitchensi* | 19 |
| *Lasioglossum laevissimum* | 23 |
| *Lasioglossum leucocomum* | 1 |
| *Lasioglossum lnewasonia* | 3 |
| *Lasioglossum macoupinense* | 3 |

| *Lasioglossum nr pilosum* | 5 |
| --- | --- |
| *Lasioglossum paravida* | 16 |
| *Lasioglossum pilosum* | 80 |
| *Lasioglossum platyparium* | 1 |
| *Lasioglossum quebecense* | 1 |
| *Lasioglossum smilacinae* | 82 |
| *Lasioglossum subviridatum* | 1 |
| *Lasioglossum versans* | 1 |
| *Lasioglossum versatum* | 3 |
| *Lasioglossum zephyrum* | 13 |
| *Lasioglossum zonulum* | 3 |
| *Sphecodes cressoni* | 3 |
| *Sphecodes dichrous* | 2 |
| Species | Total Count |
| **Megachilidae** | |
| *Anthidium manicatum* | 2 |
| *Anthidium oblongatum* | 1 |
| *Heriades carinata* | 1 |
| *Hoplitis pilosifrons* | 7 |
| *Megachile campunulae* | 4 |
| *Megachile centicularis* | 1 |
| *Megachile gemula* | 3 |
| *Megachile latimanus* | 4 |
| *Megachile montivago* | 1 |
| *Megachile relativa* | 2 |
| *Megachile rotundata* | 2 |
| *Megachile texana* | 25 |
| *Osmia pumila* | 2 |
| *Stelis lateralis* | 4 |
