# Supplementary figures and images for: B.Y.O. Bees: Managing wild bee biodiversity in urban greenspaces
Source: PLoS One. 2023 Apr 26;18(4):e0281468. doi: 10.1371/journal.pone.0281468 (PMC10132636; doi:10.1371/journal.pone.0281468)

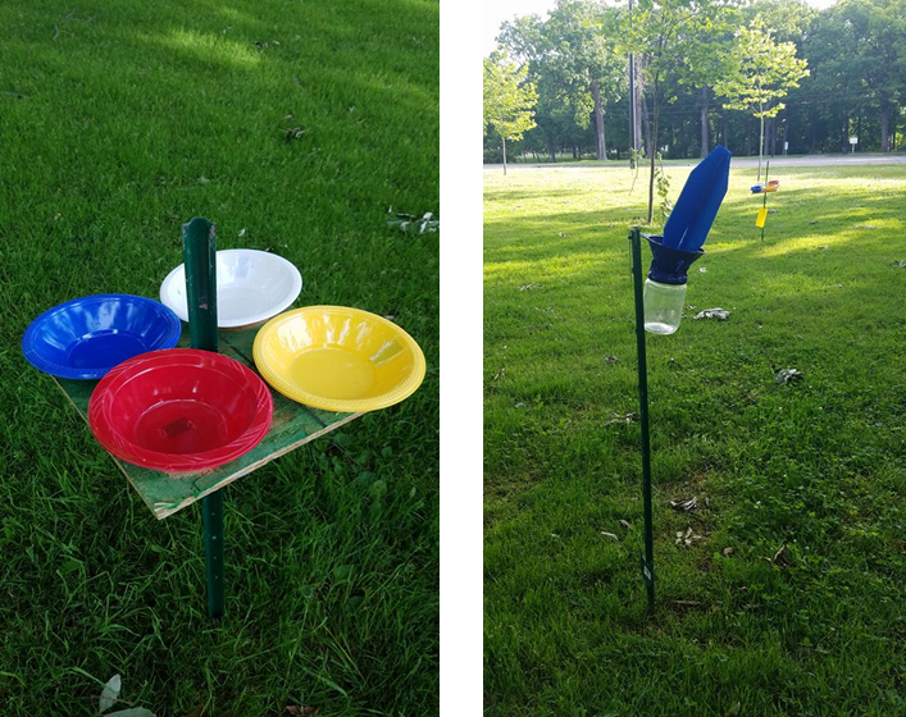

Supplement: S1 Fig — Photos of (A) a multicolored pan trap and (B) a blue vane trap at a field site. Photos: M. Anderson. (PNG) [file pone.0281468.s003.png]

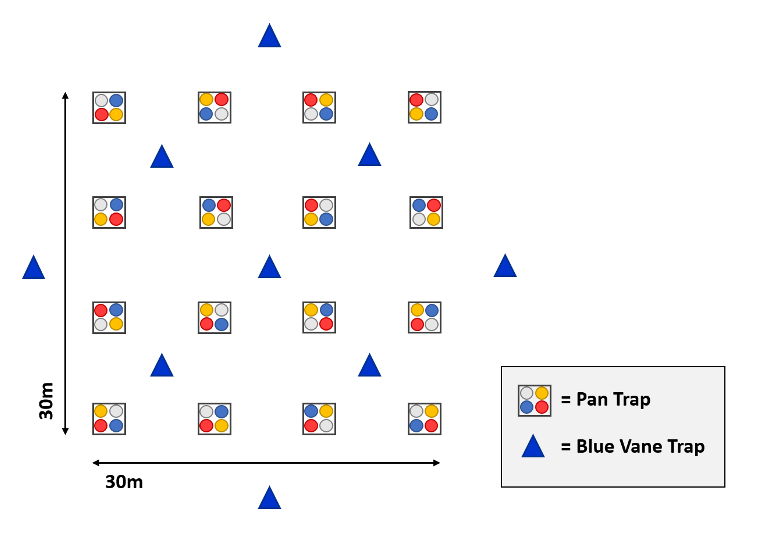

Supplement: S2 Fig — Diagram of the pan trap sample array showing the positions of multicolored pan traps and blue vane traps. (PNG) [file pone.0281468.s004.png]

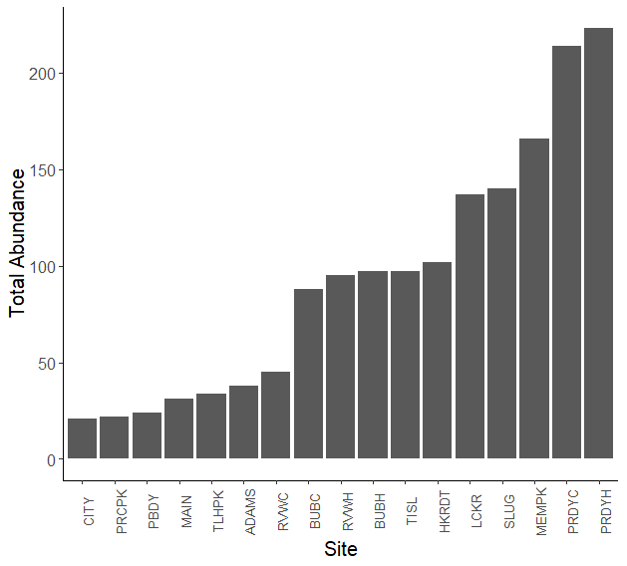

Supplement: S3 Fig — Total bee abundance observed at each site over the course of two field seasons from 2017–2018. (PNG) [file pone.0281468.s005.png]
