# Supplementary material for: B.Y.O. Bees: Managing wild bee biodiversity in urban greenspaces
Source: PLoS One. 2023 Apr 26;18(4):e0281468. doi: 10.1371/journal.pone.0281468 (PMC10132636; doi:10.1371/journal.pone.0281468)
Supplement: Attachment — Submitted filename: RevisionResponses_BYOBEEZ-MS_27July22.docx [file pone.0281468.s008.docx]

**MS title:** Response to second round of revisions for “B.Y.O. Bees: managing wild bee biodiversity in urban greenspaces”

| **Reviewer Comments** | **Author Responses** | **Notes** |
| --- | --- | --- |
| The results for the GLMs are not adequately reported. Apart from giving the estimates and AIC values for the best model, the authors should run likelihood ratio tests (LRT) to give the statistics (i.e. chi square values, df, and p values) for each variable included in the best model and report them either in a table or in the text. | We conducted two likelihood ratio tests to compare the best-fit GLMs with the global GLMs for both bee abundance and bee species richness. The output of these tests has been added as Table 2. The following sentence has also been added to the Results section starting in Line 333: “The best-fit GLMs for bee abundance and species richness were chosen according to their Akaike’s Information Criterion (AIC) values (Table 1) and their goodness-of-fit was verified using likelihood ratio tests (Table 2).” | Ran code to compute LRTs for each model (comparing original model to best-fit model as chosen via AIC). This code has been added to the main project code file. **Resolved.** |
| Line 260-263- if you just omitted them from the analysis you are comparing data from fewer pantraps there that in other places, which could bias the result. You should standardize by subsampling (Gotelli and Colwell 2001) so you have the same number of pantraps to compare everywhere, as typically done. Please either do this subsampling or clearly justify why you do not do it. It does not seem correct to compare places with different sampling effort. | Sampling effort per site was consistent, however there were more sites in certain categories compared with others. There was one more site for unmanaged urban sites (n=6), managed urban (n=5), and managed suburban (n=5). Therefore, comparisons between sites remain valid given that rarefaction was completed using the IMAX package  Trap omissions were due to animal or environmental disturbance like wind as stated in the text (l. 262-263). Trap omissions constituted less than 5% of total traps collected, and were unlikely to have significant impact on biodiversity impacts. | **Resolved** |
| add letters for significant differences after post hoc analysis in fig 2. And complete the legend with this | Figure and code have been updated to include letters | The updated figure should be uploaded along with the revisions. **Resolved.** |
| Add reference for Shannon diversity index (line 240 ) | Citation added as requested | **Resolved.** |
| Add also a reference in the sentence given in lines 67-69. | Added a reference in Line 69 to a 2020 paper by Dylewski and colleagues which finds that urban areas provide novel ecosystems for pollinators, although the effect of urban greenery on pollinators was not equal and associated with management practices. | Full paper citation: Dylewski Ł, Maćkowiak Ł, Banaszak-Cibicka W. Linking pollinators and city flora: How vegetation composition and environmental features shapes pollinators composition in urban environment. Urban forestry & urban greening. 2020 Dec 1;56:126795. |
| There is abundant literature regarding the effect of floral diversity on bee communities and it should be cited in the discussion section. Same for other parts, the discussion section lacks references to previous work. | To cite the benefits of flower diversity for pollinators in general, we included a citation in Line 385 for a 2010 paper by Fründ and colleagues which finds a strong correlation between flower diversity and visiting pollinator diversity.  We also included a citation for Woodcock et al. 2014, a paper which finds that pollinator abundance was highly correlated with legume and forb flower diversity.  For the more specific statement that overlapping blooming periods (a product of diverse flower communities) are beneficial to bee communities, we cited a 2016 paper by Venjakob and colleagues which finds that high spatiotemporal plant diversity provides more stable pollinator forage across the summer.  To demonstrate the influence of tree species diversity on bee communities, we added a reference in Line 391 to a 2009 paper by Sobek et al. which finds that forests with higher tree diversity supported greater abundances of wild bees, corroborating our findings. | Full citations:  Fründ J, Linsenmair KE, Blüthgen N. Pollinator diversity and specialization in relation to flower diversity. Oikos. 2010 Oct;119(10):1581-90.  Venjakob C, Klein AM, Ebeling A, Tscharntke T, Scherber C. Plant diversity increases spatio‐temporal niche complementarity in plant‐pollinator interactions. Ecology and evolution. 2016 Apr;6(8):2249-61.  Woodcock BA, Savage J, Bullock JM, Nowakowski M, Orr R, Tallowin JR, Pywell RF. Enhancing floral resources for pollinators in productive agricultural grasslands. Biological conservation. 2014 Mar 1;171:44-51.  Sobek S, Tscharntke T, Scherber C, Schiele S, Steffan-Dewenter I. Canopy vs. understory: does tree diversity affect bee and wasp communities and their natural enemies across forest strata?. Forest Ecology and Management. 2009 Aug 20;258(5):609-15.  **Resolved.** |
