# Supplementary material for: B.Y.O. Bees: Managing wild bee biodiversity in urban greenspaces
Source: PLoS One. 2023 Apr 26;18(4):e0281468. doi: 10.1371/journal.pone.0281468 (PMC10132636; doi:10.1371/journal.pone.0281468)
Supplement: Attachment — Submitted filename: RevisionResponses_BYOBEEZ-MS_090922.docx [file pone.0281468.s009.docx]

**MS title:** Response to second round of revisions for “B.Y.O. Bees: managing wild bee biodiversity in urban greenspaces”

**Contributors:** Maggie Anderson, Israel Del Toro, Relena Ribbons

**List of author comment and responses:**

| **Comment** | **Respondent** | **Response** | **Notes** |
| --- | --- | --- | --- |
| The results for the GLMs were not adequately reported. Apart from giving the estimates and AIC values for the best model (which one gets with the function ‘summary’), the authors should run likelihood ratio tests (LRT) to give the statistics (i.e. chi square values, df, and p values) for each variable included in the best model and report them either in a table or in the text. This can be obtained with function ‘Anova’ in library (car) or alternatively with function ‘Drop1’ applied to the best-fitting model. Otherwise we cannot know whether the variables that appear in the best model have a significant effect on the response or not. The authors have conducted two likelihood ratio tests to compare the best-fit GLMs with the global GLMs for both bee abundance and bee species richness, but this is not what I meant them to do. | MIA | The clarification of this comment was much appreciated. As suggested, we used the drop1() function in R on the model, which gave us the statistics for each variable. In the drop1() function, we specified that “test = ‘LRT’ “ to ensure that a likelihood ratio test was run. The output of these tests has been added as Table 2 in lines 301-307 | Ran code to compute LRTs for each variable of the two models. This code has been added to the main project code file. **Resolved.** |
| Another important issue is that I see that in the last revised version the authors have removed a paragraph in the statistical analyses section where the GLMs were explained. Right now, there is no explanation of the GLMs conducted. The authors should explain clearly the statistical analyses conducted (type of model, error distribution, whether it is mixed model or not, predictor and response variables) and also how did they perform model selection (did they use automatic selection with dredge function in MuMIn package? If not, did they try all the possible combinations? How did they do so?). Lastly, as commented above, the authors may add the LRT to the best-fitting model to show the significance of the variables included in it. | RRR, IDT MA | This information is included in the methods section 334-343. THis may have been inadvertently deleted in previous submissions. The GLM selection criteria and stepwise variable selection using AIC and the `mass` package. The citation and clarification is now included in text. | **Resolved** |
