# Supplementary material for: B.Y.O. Bees: Managing wild bee biodiversity in urban greenspaces
Source: PLoS One. 2023 Apr 26;18(4):e0281468. doi: 10.1371/journal.pone.0281468 (PMC10132636; doi:10.1371/journal.pone.0281468)
Supplement: Attachment — Submitted filename: ResponseToReviewersFinal.docx [file pone.0281468.s010.docx]

In our revision we addressed the final two comments: “Although the authors have included the LRT as I indicated, there are a couple of minor issues that should be solved before publication:

1) in line 339-340, the authors indicate:  'the model’s goodness-of-fit was verified using likelihood ratio tests (Table 2)'. However, LRT do no test model fit, but the significance of the effect of different predictor variables on the response. So this sentence is incorrect. The authors might rather say that the significance of the predictive variables in the best-fitting model was tested using LRT.

Author response- Line 336-337 now reads: “the R package mass (Venables and Ripley, 2002) and the significance of the predictor variables in the best-fitting model was tested using likelihood ratio tests (Table 2).”

2) Also in the new table 2 with the results of LRT, it might be 3 columns: df, Chi2 and p-values. It is fully correct to say in the legend of the table that these are the results of LRT, but in the column, the value of hte statistics is a value of Chi2 (not LRT). So please, change the heading of the column from LRT to Chi2. Also, p-values should be written adequately, using 3 decimals (or 4 if necessary), and using '< 0.0001' when p-values are smaller than 0.0001.”

Author response- Table 2 column heading has been appropriately adjusted to Chi2, and p-value significant figures were extended as suggested by this reviewer.
