# Supplementary material for: B.Y.O. Bees: Managing wild bee biodiversity in urban greenspaces
Source: PLoS One. 2023 Apr 26;18(4):e0281468. doi: 10.1371/journal.pone.0281468 (PMC10132636; doi:10.1371/journal.pone.0281468)
Supplement: Attachment — Submitted filename: PLoS ONE Reviewer Comments.docx [file pone.0281468.s007.docx]

Reviewer/Journal Comments	Author Response

| 1. Please ensure that your manuscript meets PLOS ONE's style requirements, including those for file naming. The PLOS ONE style templates can be found | We have reformatted the manuscript to comply with style requirements, thank you. |
| --- | --- |
| Please note that PLOS ONE has specific guidelines on code sharing for submissions in which author-generated code underpins the findings in the manuscript. In these cases, all author-generated code must be made available without restrictions upon publication of the work. Please review our guidelines at <https://journals.plos.org/plosone/s/materials-and-software-sharing#loc-sharing-code> and ensure that your code is shared in a way that follows best practice and facilitates reproducibility and reuse. Code may be shared by providing a URL within the Methods section to a code repository or it may be uploaded as a supplemental file.  We note that you have stated that you will provide repository information for your data at acceptance. Should your manuscript be accepted for publication, we will hold it until you provide the relevant accession numbers or DOIs necessary to access your data. If you wish to make changes to your Data Availability statement, please describe these changes in your cover letter and we will update your Data Availability statement to reflect the information you provide. | We have appended the R markdown file which includes code for these analyses. The code is also available on a Github repository: <https://www.github.com/deltoroi/BYOBEEZ>  Added to the end of manuscript (L746). |
| Please amend the manuscript submission data (via Edit Submission) to include authors Maggie Anderson, Floréal Crubaugh, Cady Greenslit, Emily Hill, Heidi Kroth, Emily Stanislawski, Relena R. Ribbons. | Amended. |
| We note that Figure 1 in your submission contain copyrighted images. All PLOS content is published under the Creative Commons Attribution License (CC BY 4.0), which means that the manuscript, images, and Supporting Information files will be freely available online, and any third party is permitted to access, download, copy, distribute, and use these materials in any way, even commercially, with proper attribution. For more information, see our copyright guidelines: <http://journals.plos.org/plosone/s/licenses-and-copyright>. | The base map was generated in R using ggmap library and a stamen basemap. Both are open access and now cited in text and references section. |
| We require you to either (1) present written permission from the copyright holder to publish these figures specifically under the CC BY 4.0 license, or (2) remove the figures from your submission: You may seek permission from the original copyright holder of Figure 1 to publish the content specifically under the CC BY 4.0 license. | We use the Stamen CC statement in the figure legend. |
| Please include a copy of Table 2 which you refer to in your text on page 8. | This was a remnant of a previous analysis that was replaced by the GLM table. This text is now omitted. |
| The [PLOS Data policy](http://www.plosone.org/static/policies.action#sharing) requires authors to make all data underlying the findings described in their manuscript fully available without restriction, with rare exception (please refer to the Data Availability Statement in the manuscript PDF file). The data should be provided as part of the manuscript or its supporting information, or deposited to a public repository. For example, in addition to summary statistics, the data points behind means, medians and variance measures should be available. If there are restrictions on publicly sharing data—e.g. participant privacy or use of data from a third party—those must be specified. | These data are fully available in the Github repository (referenced at the end of the main manuscript file): see L746.  https://www.github.com/deltoroi/BYOBEEZ |
| This paper presents the results of a two-year research evaluating bees in urban and suburban habitats. Urban habitats were both managed and unmanaged ones. Whereas, suburban habitats were exclusively the managed ones. Management was found to increase bee richness and abundance. In my view, this is an overstatement as the unmanaged suburban areas were not subject to an analysis. Thus, this conclusion needs to be made more precise. | We clarify that no unmanaged suburban sites are included in this analysis because we could not secure permits in county parks. We adjust our conclusions to keep within the scope of the paper. We acknowledge this limitation throughout the conclusion section and identify it as an area of key future investigation. L491-495:  We do note that our results do not reflect potential diversity patterns in unmanaged suburban sites. Future research could address the relationship between pollinator diversity and traditionally managed landscapes that are not focused on pollinators; thus, adding a missing piece of knowledge about these unmanaged suburban sites into pollinator biodiversity assessments. |
| Management regimes and landscape scale. Was there a management level, i.e., what % of the area was sown with pollinator-friendly plants? What % of the area was designated to fulfil this criterion? Could you briefly describe what plant species were dominant in the unmanaged habitats? Please, explain why unmanaged suburban areas were not included in the research conducted. | We added details on the percent cover of area needed to achieve managed designation in lines 159-171. |
| Bee sampling and identification. Information on the total number of samples taken in each year of the study and in the urban and suburban habitats would be useful. | Urban subset: 1008 samples.Urban managed subset: 840 samples. Suburban managed: 780 samples. This information isadded in L292-294. |
| For the purpose of giving other scientists an idea of the climatic conditions in the research area, please add information on what average temperatures prevail in the area during springtime and summertime period. | Details are added in Lines 134-137. |
| Each site was sampled with the pan trap array once every two weeks..... Define precisely what the trial means to you. The sum of bees caught in all bowls? Was this data merged? | A single sampling unit is equivalent to one bowl/one pant trap. “Each pan was treated independently as its own sample.” added in line 191.  Data was not merged, so rarefaction analysis could be completed. |
| Analyses Tree species diversity was assessed at each site within a 20m x 20m plot contained within the 30m x 30m pan trap array plot .....  Were the flower colours of woody plants also assessed with the use of colour wheel? Why no assessment of the bee nutrient base in the springtime was conducted? Could you additionally include a list of plants with their assigned flower colours in the appendix? | No woody flower colors were assessed with a colour wheel.  We have added a supplementary list of plant species and assigned colours as well as locations “Floral Diversity Supplementary.” |
| Bee community response to management styles.  Formulate your conclusions carefully. The sentence above may be misleading as the unmanaged suburban habitats were not included in the research. | We have now added “Please note there were no unmanaged suburban sites for comparison, so unmanaged sites only refer to unmanaged urban areas.” to line 365, and reiterate this point in lines 439-443. |
| Discussion Although some landscape-level variables were positively associated with wild bee ... What is the plants base in the springtime like? I am missing this information. | We have added a file of the floral diversity when we sampled, but only have anecdotal information or data from other studies on plant base in the spring time since that was not the time period for our study. |
| Recommendations for greenspace management.  You should also refer your results to the literature which indicates that the unmanaged habitats are also attractive to wild bees. Unmanaged sites, rich in the floral species also provide stable resources.  Twerd L., Banaszak-Cibicka W. 2019. Wastelands: their attractiveness and importance for preserving the diversity of wild bees in urban areas. Journal of Insect Conservation 23(3): 573–588. Machon N. 2021. Urban Wastelands Can Be Amazing Reservoirs of Biodiversity for Cities. Urban wastelands 3-18. McKinney. 2021. Strategies for Increasing Biodiversity Conservation in Cities Using Wastelands: Review and Case Study. Urban wastelands 39-64. Twerd L., Banaszak-Cibicka W., Sobieraj-Betlińska A., Waldon-Rudzionek B., Hoffmann R. 2021. Contributions of phenological groups of wild bees as an indicator of food availability in urban wastelands. Ecological Indicators 126: 107616. | We have now added the following to L449-451.  Work by Twerd and Banaszak‑Cibicka (2019) found “urban wastelands” provided important secondary habitats for bees, where grassy suburban unmanaged sites and reclaimed sand and clay pits were especially attractive habitats for bees. |
| Reviewer 2: My primary concerns with this study have to do with the study design. The paper lacks specific information on methodology. More details about the study sites should be provided. It could be done in the table or in a more descriptive manner. What were the management practices for pollinators, floral diversity, tree diversity on individual sites? | We describe the management definitions in the second section of the methods “Management regimes and landscape scale”. Managed sites were those that used pollinator friendly seed mixes and/or created nesting habitat for native pollinators. |
| Justification of three study site types selection (managed urban sites, unmanaged urban sites and managed suburban sites) is needed. Why are unmanaged suburban sites missing? | We agree that unmanaged suburban sites would be beneficial for the study design, but were unable to secure permission from private landowners who were not engaged with management. These sites are managed by County government and they did not grant permission for sampling in their greenspaces. |
| How many samples were collected for each site type? | 8 sampling dates per site, between 2017 and 2018. 21 traps per site, for n=168 independent observations per site. Now added to L291-292. |
| Add reference to the method floral diversity evaluation at each site. | See L262-269, with the amendment of “using dichotomous keys in Wildflowers of Wisconsin and the Great Lakes Region: A Comprehensive Field Guide” too. |
| There are no figures for PCoA analyzes. | The PCoA values are used as regression predictors in the model, so a standalone figure isn’t necessary. The key findings are summarized in text. |
| Information on bee diversity is missing in the results, but appears in the discussion for the first time in the paper. | We have added a subheading to the results section, to signpost results on diversity (richness metrics). |
| “Although habitat fragmentation in urban areas negatively affects bee diversity, we demonstrate that many of these effects can be remedied through the active management greenspaces for wild bees.” - was it analyzed at this paper? I don’t believe that such analyzes are mentioned in the methods section. | We have added the following to L490-494:  This is consistent with a meta-analysis by Winfree et al. (2009) who found that habitat fragmentation significantly negatively influenced bee populations, when in systems with little natural habitat remaining. Urban spaces managed for pollinator conservation can alleviate this negative influence, by providing a variety of habitat and floral resources for bees, which is supported by this study and other recent work on urban bee populations (Del Toro and Ribbons, 2020; Lerman and Milam, 2016).  We have added the two references as well. |
